# Immediate effect of caffeine on sympathetic nerve activity: why coffee is safe? A single-centre crossover study

**DOI:** 10.1007/s10286-023-00967-5

**Published:** 2023-08-20

**Authors:** Jennifer M. Butler, Christopher M. Frampton, Grant Moore, Murray L. Barclay, David L. Jardine

**Affiliations:** 1 Department of General Medicine, Christchurch Hospital, 2 Riccarton Ave, Private Bag 4710, Christchurch, New Zealand; 2 Department of Medicine, Christchurch School of Medicine, University of Otago, Dunedin, New Zealand; 3 Department of Toxicology, Christchurch Hospital, 2 Riccarton Ave, Christchurch, New Zealand; 4 Department of Clinical Pharmacology, Christchurch Hospital, 2 Riccarton Ave, Christchurch, New Zealand

**Keywords:** Caffeine, Muscle sympathetic nerve activity, Cardiovascular system, Adenosine

## Abstract

**Purposes:**

Habitual coffee drinking is ubiquitous and generally considered to be safe despite its transient hypertensive effect. Our purpose was to determine the role of the sympathetic nervous system in the hypertensive response.

**Methods:**

In a single-centre crossover study, medical caregivers were studied after consumption of standard coffee (espresso), water and decaffeinated coffee (decaff) given in random order at least 1 month apart. Plasma caffeine levels, mean arterial pressure, heart rate, total peripheral resistance and muscle sympathetic activity were recorded. Baroreflex activity was assessed using burst incidence and RR interval changes to spontaneous blood pressure fluctuations.

**Results:**

A total of 16 subjects (mean [± standard error] age 34.4 ± 2 years; 44% female) were recruited to the study. Three agents were studied in ten subjects, and two agents were studied in six subjects. Over a 120-min period following the consumption of standard coffee, mean (± SE) plasma caffeine levels increased from 2.4 ± 0.8 to 21.0 ± 4 µmol/L and arterial pressure increased to 103 ± 1 mmHg compared to water (101 ± 1 mmHg; *p* = 0.066) and decaff (100 ± 1 mmHg; *p* = 0.016). Peripheral resistance in the same period following coffee increased to 120 ± 4% of the baseline level compared to water (107 ± 4; *p* = 0.01) and decaff (109 ± 4; *p* = 0.02). Heart rate was lower after both coffee and decaff consumption: 62 ± 1 bpm compared to water (64 bpm; *p* = 0.01 and *p* = 0.02, respectively). Cardio-vagal baroreflex activity remained stable after coffee, but sympathetic activity decreased, with burst frequency of 96 ± 3% versus water (106 ± 3%; *p* = 0.04) and decaff (112 ± 3%; *p* = 0.001) despite a fall in baroreflex activity from – 2.2 ± 0.1 to – 1.8 ± 0.1 bursts/100 beats/mmHg, compared to water (*p* = 0.009) and decaff (*p* = 0.004).

**Conclusion:**

The hypertensive response to coffee is secondary to peripheral vasoconstriction but this is not mediated by increased sympathetic nerve activity. These results may explain why habitual coffee drinking is safe.

**Supplementary Information:**

The online version contains supplementary material available at 10.1007/s10286-023-00967-5.

## Introduction

Coffee is the second most popular beverage in the world, (after tea) and has been used for its stimulatory effects for at least 500 years. The feeling of arousal is secondary to caffeine blocking the A_1_ and A_2a_ adenosine receptors in the cortex and basal ganglia of the brain [1]. In the periphery, caffeine also blocks the inhibitory effects of adenosine on the myocardium, blood vessels and sympathetic nerves [2, 3]. This may cause increased cardiac output (CO), vasoconstriction or both, and therefore acute hypertension. The dominant mechanism is thought to be peripheral vasoconstriction, although exactly where this takes place is uncertain [4–6]. Some indices of sympathetic activity, including serum catecholamines and renin, increase rapidly in response to caffeine but not enough to stimulate vasoconstriction or CO [7–11]. Measuring central sympathetic responses to drugs is difficult because any agent that increases blood pressure (BP) acutely will also trigger increased baroreflex feedback which in turn inhibits sympathetic nerve activity (SNA) [12, 13]. Muscle sympathetic nerve activity (MSNA) is currently the most reliable dynamic indicator of SNA, but to our knowledge there is only one previous study documenting immediate responses to coffee, with the authors reporting that the hypertensive response to coffee was associated with an immediate increase in MSNA levels [14]. This result suggested that a central effect of caffeine (or some other component of coffee) could overcome baroreflex-mediated inhibition of MSNA.

We therefore wanted to clarify the relationship between coffee ingestion, caffeine levels, BP, CO and SNA. We postulated that the hypertensive effect of coffee is caffeine mediated and so would be greater after consumption of a standard espresso than after consumption of decaffeinated coffee (decaff) or water. We also predicted that the baroreflex mechanism might acutely decrease MSNA in response to an increase in BP.

## Methods

The study was approved by the National Ethics committee. Written informed consent was obtained from all subjects prior to taking part in the study.

### Subject selection

Over a 6-month period, subjects were selected exclusively from healthcare providers working in our hospital who answered a local advertisement. To be selected, subjects had to have a moderate daily caffeine intake in the form of coffee, tea, energy drinks, chocolate among others. The overall aim of subject selection was for the study to be applicable to the general caffeine-using population but to avoid excessive variation in dose–response between subjects such as, for example, tolerance in those with regular high caffeine intake, as well as exaggerated responses in caffeine-abstainers [4–6, 9]. We selected non-obese, non-smoking subjects of a similar age with minimal medical morbidities or medications that would potentially affect caffeine metabolism [15].

### Coffee preparation

Coffee was prepared by passing water under pressure through ground coffee beans using a standard espresso machine. The coffee was drunk from a paper cup in a volume of 100 ml. Decaff was prepared using the espresso machine but with beans decaffeinated by the dichloromethane solvent technique [16]. The subjects and the investigators were blinded as to whether coffee or decaff was given. Water (100 ml) was served at room temperature. Low volumes were used to help ingestion in the supine position and avoid any other effects on blood volume or osmoreceptors.

### Caffeine measurements

Measurements of caffeine in plasma were performed using an in-house high-performance liquid chromatography assay developed and validated by Toxicology, Canterbury Health Laboratories, Christchurch (New Zealand). Briefly, the assay used liquid–liquid extraction for sample preparation and β-hydroxyethyl theophylline as the internal standard (see Electron Supplementary Material). The lower limit of quantification for the assay was 0.30 umol/L and the coefficient of variation was < 10%.

### MSNA and haemodynamic measurements

Microneurography was performed on the right superficial peroneal nerve [17]. The nerve signal was amplified (× 50,000), filtered (700–2000 Hz), integrated (time constant 0.1 ms) and displayed on-line with the BP and electrocardiogram. Nerve bursts were counted (by a microneurographer who was blinded to the agent) provided that the following criteria were met: the signal-to-background ratio was > 3; the bursts were pulse-synchronous; burst amplitude was inversely proportional to diastolic BP; and skin activity was absent [18]. In addition, we also quantified MSNA burst frequency using an automated program which identified bursts according to their shape and time parameters (see sections Sympathetic baroreflex activity and Cardiovagal baroreflex activity). Non-invasive beat-to-beat BP was measured from the middle phalanx of the right middle finger (Finometer Blood Pressure Monitor; Finapres Medical Systems B.V., Amsterdam, The Netherlands) with the hand held at heart level. Beat-to-beat changes in stroke volume (SV) was estimated by modelling flow from the arterial pressure waveform (Modelflow; TNO Biomedical Instrumentation, Amsterdam, The Netherlands) [19]. CO was derived from SV × HR, and total peripheral resistance (TPR) was calculated from mean arterial pressure (MAP) divided by CO.

### Sympathetic baroreflex activity

Burst incidence was measured by the threshold analysis technique for sympathetic baroreflex sensitivity. We used local purpose-built software for analysing the MSNA-MAP correlation over time intervals of 180 s. All intervals were visually inspected. We rejected parts of the MSNA recordings distorted by interference or associated with unsatisfactory BP waveforms (e.g. Finapres calibrations). A representative MSNA burst was chosen and profiled for amplitude, duration and latency. In this way, the program constructed a specific time window during which a nerve burst of acceptable amplitude would be selected for baroreflex analysis. For each time interval of 180 s, bursts were then automatically selected by the program, counted and placed in 3-mmHg bins. Burst incidence was calculated for each bin and then plotted against MAP to construct a baroreflex curve [20, 21]. Between three and ten curves were constructed from data pooled over the 30-min time interval preceding each time point: baseline, and 60 and 120 min. We reasoned that because the subjects were in a supine position, spontaneous BP fluctuations would be relatively small and close to the operating point (on the linear part of the curve). The limiting factors for calculating slopes were the quality of the nerve field, the amplitude of spontaneous BP fluctuations and MSNA burst frequency. Individuals with lower burst frequencies had lower burst counts in all BP bins and therefore fewer coordinates on their regression slopes.

### Cardiovagal baroreflex activity

Cardiovagal baroreflex sensitivity was measured by calculating the slope of the regression line between linearly related changes in systolic BP and pulse interval over four or more consecutive beats. Slopes were accepted provided pulse interval and systolic BP changed by more than 5 ms and 1 mmHg, respectively (*R*^2^ > 0.80) [22]. Values were averaged over 30-min time intervals preceding baseline, 60 and 120 min.

### Protocol

Subjects were instructed to abstain from all forms of caffeine for 24 h before the study. All studies were conducted between 9.00 am and 12.00 pm. Recording sessions for each agent (coffee, water or decaff given in a random order) were at least 1 month apart. Subjects were positioned in a supine position. BP, HR and microneurographic monitoring was started and continued until the measurements were stable over a 30-min period. Baseline measurements were taken. After this, the subjects were administered either water, coffee or decaff, via a straw from the paper cup, and recordings were continued for a further 120 min.

### Time intervals and averaging

#### Caffeine levels

For each treatment, the area under the curve was calculated using all nine time points (baseline, and 15, 30, 45, 60, 75, 90, 105 and 120 min). Comparisons were made after logarithmic transformation of the areas using analysis of variance (ANOVA).

#### Haemodynamics and MSNA indices

Initially we averaged haemodynamic variables MAP, HR, SV, CO, TPR and MSNA indices over 5-min periods at time intervals of 15 min, namely baseline and 15, 30, 45, 60, 75, 90, 105 and 120 min. Mean values from the first four time points (15, 30, 45, 60 min) and the second four time points (75, 90, 105, 120 min) were averaged again to constitute the two target time points: 60 and 120 min.

For Pearson correlation studies between haemodynamic variables and caffeine levels we used the values at baseline and the initial 15-min time interval from the first 60 min and averaged the 15-min values from the second 60 min for the last time point (6 time points in total).

### Statistics

For MAP and HR, comparisons were made using absolute values with baseline as a covariate. For SV, CO, TPR and MSNA variables, comparisons were made using the percentage of baseline values. Between-group effects for time and treatment (water, coffee or decaff) were assessed initially, followed by pairwise comparisons using *t*-tests when the ANOVA *p* was significant. When pairwise comparisons demonstrated a difference between treatments, individual time-point comparisons were made. For sympathetic and cardiovagal baroreflex activity, comparisons between groups were made at baseline and at 60 and 120 min using absolute values.

## Results

A total of 16 subjects were studied, with complete data obtained for all three agents (water, coffee and decaff) in ten subjects. In the remaining six subjects, only two agents were studied. The mean (± standard error [SE]) age of subjects was 34.1 ± 2.5 years, and gender distribution was equal (44% female) (Table 1). All subjects were healthy, non-smoking adults who ingested only moderate amounts of caffeine daily.

**Table 1:** Demography, caffeine intake and baseline data for the 16 subjects in the study

Characteristics of study population	Values^a^		
Demography			
Age, mean (SE), years	34.4 (2)		
Gender (male/female),* n*	9/7		
Body mass index, mean (SE)	24.5 (3)		
Daily caffeine intake			
Coffee, mean (SE) cups	2 (0.3)		
Tea, mean (SE) cups	1.5 (0.2)		
Other caffeine drinks, mean (SE),* n*	1.0 (0.1)		
Medical conditions, *n*			
Asthma	2		
Oesophageal reflux	2		
Psioriatic arthritis	1		
Endometriosis	1		
Medications, *n*			
Omeprazole	2		
Methotrexate	1		
Steroid inhalers	2		
Baseline data, mean (SE)	Water	Coffee	Decaffeinated coffee
Mean arterial pressure, mmHg	94 (3)	96 (3)	97 (4)
Heart rate, beats/min	62 (2)	64 (2)	64 (2)
MSNA, bursts/min	22 (2)	26^b^ (2)	22 (2)
MSNA, bursts/100 beats	36 (4)	40^b^ (4)	35 (3)
Cardiovagal baroreflex, mmHg/s	13.6 (1)	11.5 (1)	11.9 (1)
Sympathetic baroreflex, bursts/100 beats/mmHg	– 2.1 (0.1)	– 2.2 (0.1)	– 2.0 (0.1)
Serum caffeine concentration, mean (SE) µmol/L	3.9 (1)	2.4 (1)	5.3 (1)
Caffeine dose, mean (SE) mmol		1.1 (0.1)	0.1 (0.01)

*MSNA* Muscle sympathetic nerve activity,* SE* standard error

^a^Values are presented as the mean with the SE in parentheses, or as frequency (*n*), as appropriate

^b^Paired *t*-test difference between coffee and both other agents (*p* = 0.03 and *p* = 0.05)

### Baseline measurements

Baseline levels of MAP and HR were similar. MSNA burst frequency and incidence were higher before coffee consumption than before water and decaff consumption (*p* = 0.03 and *p* = 0.05, respectively). We found that the mean (± SE) caffeine dose in a standard cup of espresso coffee was 1.1 ± 0.1 mmol (approximately 200 mg) compared to 0.1 ± 0.01 mmol in decaff coffee. Serum caffeine levels were similar at baseline in all groups (but not zero) despite abstinence for 24 h before the study (see section Protocol).

### Caffeine levels and haemodynamic responses

**Fig. 1:**
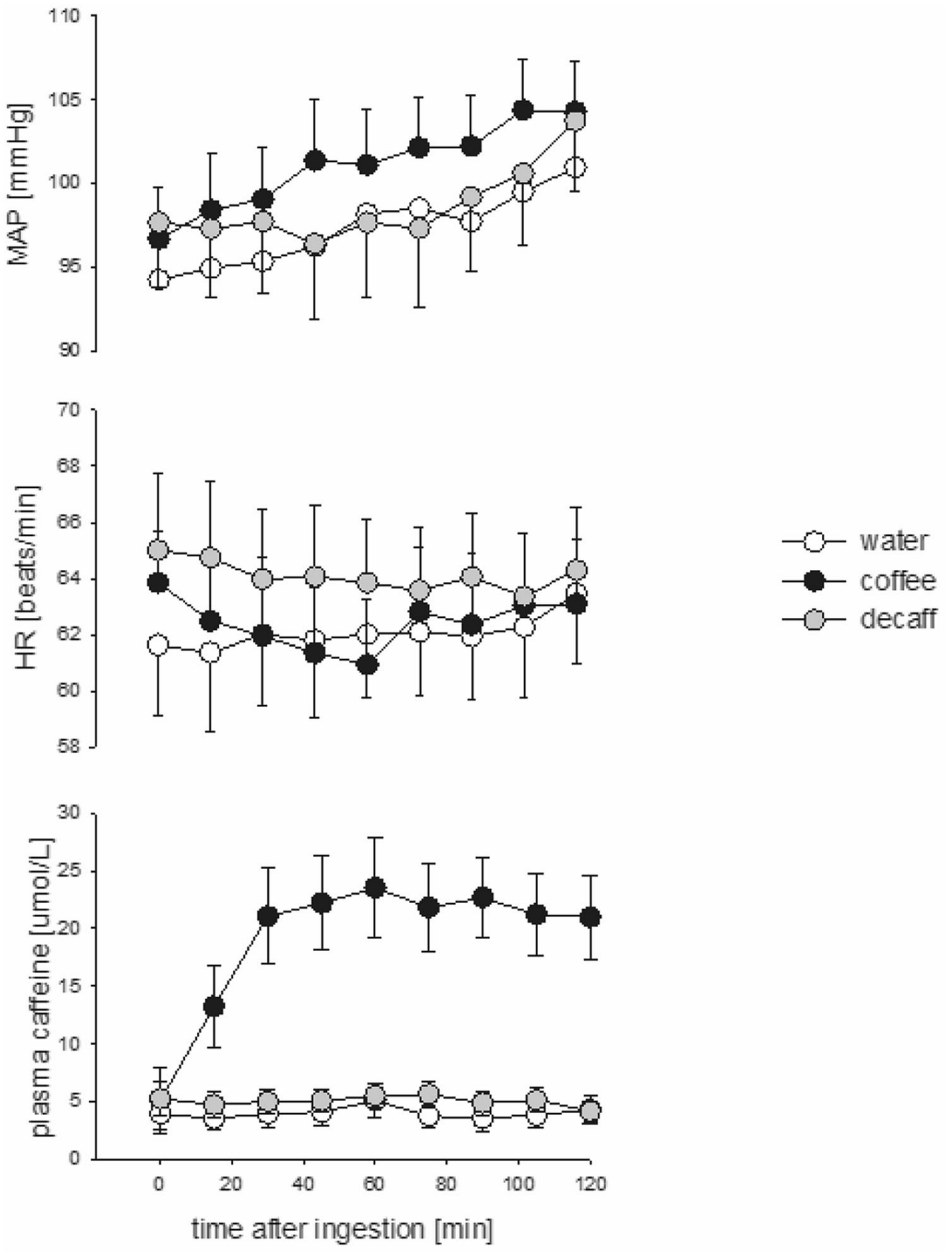
Mean arterial pressure (*MAP*), heart rate (*HR*) and plasma caffeine levels after consumption of water, coffee and decaffeinated coffee (*decaff*) during the study period

**Fig. 2:**
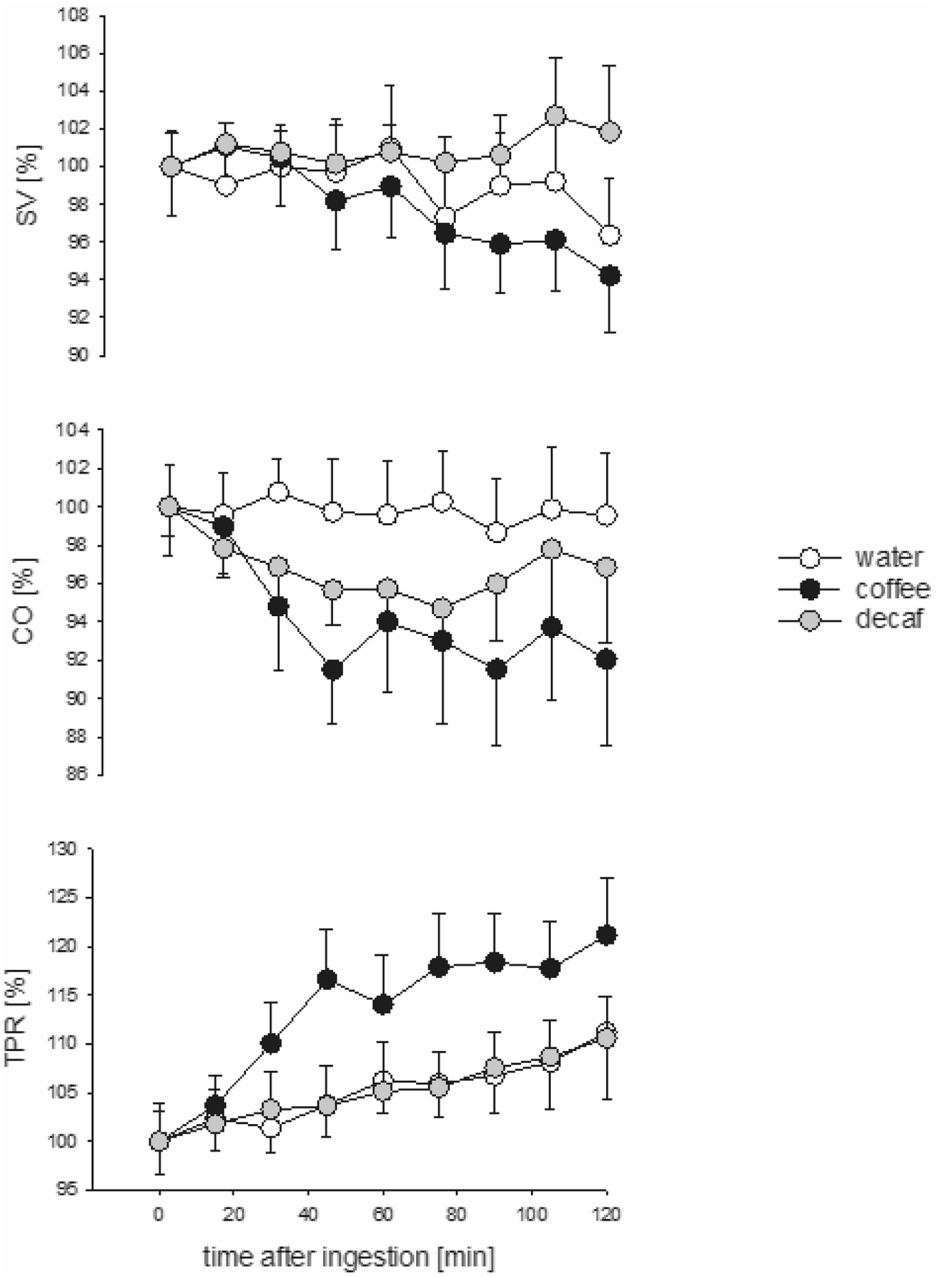
Percent baseline stroke volume (*SV*), cardiac output (*CO*) and total peripheral resistance (*TPR*) after consumption of water, coffee and decaffeinated coffee (*decaff*) during the study period

Plasma caffeine levels remained low after the consumption of water and decaff, with mean (± SE) baseline values of 3.9 ± 1 and 5.3 ± 1 µmol/L, respectively. Over the next 120 min these levels remained stable at 4.3 ± 1 and 4.2 ± 1 µmol/L; respectively (Fig. 1). The area under the curve analysis demonstrated higher plasma levels after the consumption of decaff compared to water (*p* = 0.03). After coffee, plasma caffeine levels increased by tenfold, from the baseline level of 2.4 ± 0.8 µmol/L to 21.0 ± 4 µmol/L at 120 min. Despite the differences in caffeine levels, MAP increased progressively after all three agents (ANOVA, *p* = 0.002), but it was higher (averaged over 4 time points) after coffee at 60 and 120 min (101 ± 1 and 103 ± 1 mmHg, respectively) compared to water (98 ± 1 and 101 ± 1 mmHg, respectively; *p* = 0.066) and decaff (97 ± 1 and 100 ± 1 mmHg, respectively; *p* = 0.016). The increase in MAP after coffee correlated to the plasma caffeine levels (*R* = 0.6, *p* = 0.02). After coffee and decaff consumption, HR (corrected for baseline levels) remained lower at both time points compared to water: 61 ± 1 and 62 ± 1 bpm, respectively, compared to 64 ± 1 bpm; *p* = 0.01 and *p* = 0.02, respectively). There was no correlation between HR and caffeine levels (*R* = – 0.5, *p* = 0.1). SV and CO decreased after coffee consumption at both time points (99 ± 2% and 95 ± 2%, respectively, and 95 ± 3% and 93 ± 3%, respectively) but no more so than after water and decaff (*p* = 0.1 and *p* = 0.06, respectively) (Fig. 2). However, TPR increased after coffee to 112 ± 4 at 60 min and 120 ± 4% at 120 min compared to water (102 and 107 ± 4%, respectively; *p* = 0.005) and decaff (104 and 109 ± 4%, respectively; *p* = 0.015) (Fig. 2).

### Sympathetic nerve activity

**Fig. 3:**
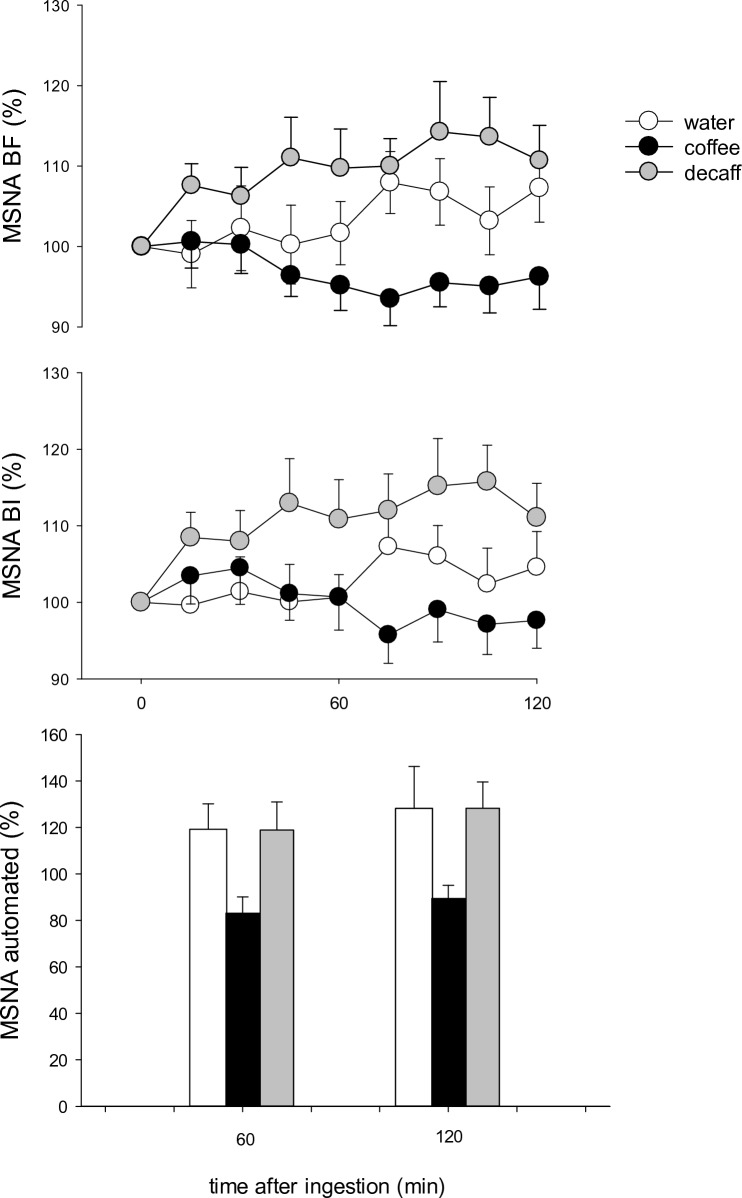
Burst frequency (*BF*; bursts/min; upper panel) and burst incidence (*BI*; bursts/100 beats; middle panel) after consumption of water, coffee and decaffeinated coffee (*decaff*) during the study period. Lower panel shows automated muscle sympathetic nerve activity (*MSNA*) BF 60 and 120 min after the consumption of water, coffee and decaf

Muscle sympathetic nerve activity was decreased at both time points after the consumption of coffee compared to water and decaff consumption. Burst frequency (% baseline) decreased to 99 ± 3% and 95 ± 3, compared to water (101 ± 3% and 106 ± 3%; *p* = 0.04) and decaff (109 ± 3% and 112 ± 3%; *p* = 0.001) (Fig. 3). Burst incidence was similar at both time points after coffee and water: 102 ± 3% and 97 ± 3 compared to 99 ± 3 and 104 ± 3 (*p* = 0.53); but it was less than that of decaff: 111 ± 3% and 115 ± 3% (*p* = 0.001). During the modest increase in MAP after decaff consumption, there was no evidence of MSNA inhibition; in fact, burst frequency increased compared to water (burst frequency *p* = 0.02, burst incidence *p* = 0.001). Automated measures of MSNA burst frequency demonstrated a clear fall to 91 ± 11% baseline after coffee compared to water (128 ± 11%; *p* = 0.002) and decaff (132 ± 11%; *p* = 0.001). These differences were apparent at both the 60 and 120 min time points: coffee versus water (*p* = 0.01 and 0.04) and coffee versus decaf (*p* = 0.006 and *p* = 0.02). Automated measures demonstrated that MSNA responses to decaff and water were similar (*p* = 0.9) (Fig. 3).

### Baroreflex activity

**Fig. 4:**
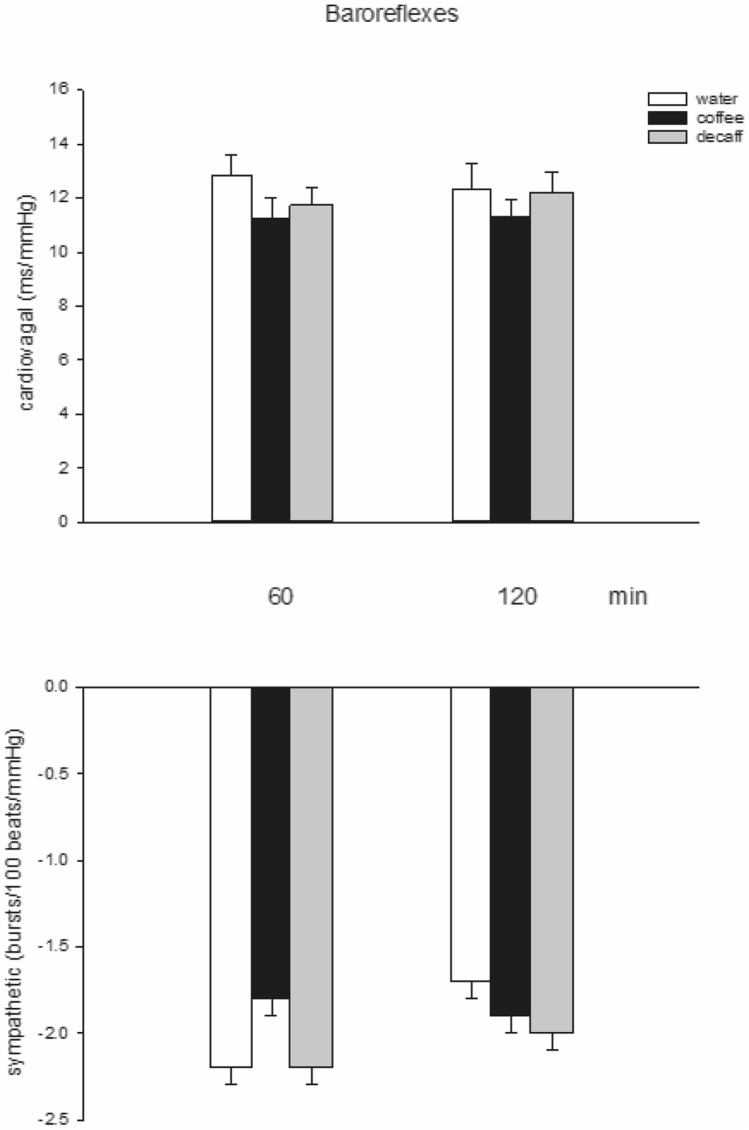
Cardiovagal and sympathetic (threshold) baroreflex sensitivity 60 and 120 min after the consumption of water, coffee and decaffeinated coffee (*decaff*)

Over the first 60 min, sympathetic baroreflex activity (threshold technique) decreased from – 2.2 ± 0.1 bursts/100 beats/mmHg to – 1.8 ± 0.1 after coffee consumption compared to water and decaff consumption; both of the latter had no clear effect: – 2.1 ± 0.1 bursts/100 beats/mmHg to – 2.2 ± 0.1 (*p* = 0.009) and – 2.0 ± 0.1 bursts/100 beats/mmHg to – 2.2 ± 0.1 (*p* = 0.004, respectively. At the same time, cardio-vagal baroreflex activity remained constant after all three agents: 11.8 ± 1 ms/mmHg to 11.3 ± 1 after coffee compared to 13.7 ± 1 ms/mmHg to 13.0 after water and 11.8 ± 1 ms/mmHg to 11.6 after decaff (ANOVA, *p* = 0.7 for time effects, 0.06 for treatment and 0.8 for time-treatment interaction) (Fig. 4).

## Discussion

As expected, consumption of a cup of espresso containing approximately 1 mmol (or 200 mg) of caffeine caused a progressive tenfold increase in plasma caffeine levels, most of which occurred during the first 60 min. This increase was associated with peripheral vasoconstriction and a modest increase in MAP (approximately 7 mmHg over 120 min). Simultaneously, there was a minor decrease in HR mediated by stable cardiovagal baroreflex activity. Lesser hypertensive effects were seen after decaff. Most importantly, the increase in MAP after coffee did not appear to be mediated by increased CO or sympathetic vasoconstrictor activity (MSNA) in skeletal muscle. Instead, CO tended to fall and MSNA clearly decreased after coffee ingestion, consistent with baroreflex-mediated inhibition. Therefore, the vasoconstriction we demonstrated after coffee consumption was probably secondary to direct blockade of adenosine receptors in peripheral vascular beds, not to sympathetic-mediated vasoconstriction in skeletal muscle. In addition to these peripheral effects, we found a damping of sympathetic baroreflex sensitivity, consistent with central stimulation of the brainstem by caffeine. Caffeine ingestion causes a modest increase in BP associated with baroreflex inhibition of the HR and MSNA. However, there is also central alteration of sympathetic baroreflex control, presumably part of the direct stimulatory effect of caffeine on the brain.

Most haemodynamic studies have demonstrated a modest increase in BP within 60 min of coffee or caffeine ingestion [4–6]. There is general agreement that despite minor surges in circulating catecholamines and renin, increased CO is not the likely mechanism for this response [15, 23–25] and rather it is more likely to be secondary to caffeine-induced vasoconstriction [23, 26, 27]. The mechanism for this is thought to be blockade of peripheral vasodilatory adenosine receptors by circulating caffeine [1, 2]. Accordingly, most studies have demonstrated a fall in CO [15, 23, 25, 27, 28]. Exactly where the vasoconstriction occurs is uncertain because nearly all forearm plethysmography studies have shown stable blood flow to skeletal muscle after caffeine consumption [10, 11, 29–32]. More recently, Corti et al. found an approximate 10% increase in MSNA burst rate after both intravenous caffeine and oral coffee, consistent with sympathetic-medicated vasoconstriction in skeletal muscle [14]. Unfortunately, these authors did not perform plethysmographic recordings. Our demonstration of decreased MSNA after coffee ingestion is more in agreement with the results of previous plethysmographic studies, all of which demonstrated no change in forearm blood flow. Coffee did not appear to stimulate MSNA. Rather, MSNA was decreased, secondary to inhibition of the caudal ventro-lateral medulla by the arterial baroreflex pathway in response to increased BP. A similar baroreflex mechanism was also responsible for the mild bradycardic response we (and others) observed after coffee consumption, namely stimulation of the nucleus ambiguous (also in the caudal ventro-lateral medulla). The lack of correlation between caffeine levels and HR is more consistent with a baroreflex mechanism than with a direct effect (of caffeine) on the sinus node. Based on our own data, we think it unlikely that caffeine has any central effect on cardiovagal baroreflex activity [33, 34]. However, we did observe evidence for a damping effect of caffeine on sympathetic baroreflex activity, which potentially might decrease the inhibition of MSNA by the baroreflexes. Against this should be weighed the effects of preserved cardiovagal baroreflex activity on MSNA levels. Any degree of bradycardia has the potential to decrease MSNA burst frequency because sympathetic bursts are closely gated to heart beats [17]. Therefore, peripheral vasoconstriction in response to caffeine may be modulated to some extent by HR and central effects on sympathetic baroreflex function at brainstem level.

The caffeine dose we measured in coffee (approx. 1 mmol or 200 mg) was similar to the amounts used previously in multiple studies [4–6]; accordingly plasma caffeine levels were also similar [14, 15, 23, 28, 31, 32]. However, the dose of caffeine in our decaff preparation was approximately 0.1 mmol (20 mg), which is fivefold higher than what the manufacturers claim and what has been quoted in other studies [10, 16, 23, 35, 36]. As such, the peak plasma caffeine levels we measured after consumption of decaff were approximately double those been previously studied: (approx. 4 vs. 2 µmol/L). Not surprisingly, we saw some “caffeine effect” after the consumption of decaff; for example, a minor increase in BP and a decrease in HR. It seems more likely that these effects (previously demonstrated in non-habitual coffee drinkers) are secondary to “residual” caffeine (not extracted by the solvent technique) rather than being caused by another vasoactive substance not extracted by the decaffeination process [14]. We are uncertain as to why manual measures of MSNA demonstrated higher levels after decaff consumption compared to water. This may relate to decaff having a lesser hypertensive effect (and therefore less baroreflex inhibition of MSNA). Another possibility would be that at low plasma caffeine concentrations, brainstem stimulation may overide baroreflex inhibition of MSNA; however, we did not find any evidence for damping of sympathetic baroreflex activity after the consumption of decaff. We accept that MSNA, although representative of SNA, may not necessarily correlate to activity in other vascular beds. For example, adrenaline levels have been shown to double in previous studies although the exact mechanism for this is uncertain [8, 10, 32]. Therefore if MSNA is stable or decreased, BP might be increased by SNA directed to, for example, renal, splanchnic and hepatic vessels [37].

In summary, the hypertensive response to coffee is secondary to peripheral vasoconstriction, but this is not mediated by MSNA and therefore probably not in muscle. Instead, there is appropriate baroreflex-mediated inhibition of MSNA and stimulation of the vagus, resulting in slowing of the HR. Therefore, the vasoconstriction we observed is more likely secondary to direct blockade of adenosine receptors by caffeine rather than sympathetic-mediated vasoconstriction in skeletal muscle. MSNA levels fell even though we observed some damping of baroreflex activity, which is presumably part of the central effect of caffeine on the brain. It may be that baroreflex-mediated inhibition of SNA is part of the explanation for the reported safety of coffee despite its stimulatory effects on the brain [4–6, 37]. The transient hypertensive effect we recorded was modest, consistent with previous studies [4–6]. Presumably the magnitude of the hypertensive response is limited by the baroreflex mechanisms we have reported [37]. Furthermore, long-term studies have not demonstrated chronic hypertension in response to habitual caffeine consumption, probably because tolerance to caffeine develops rapidly and is sustained [4–6, 9]. The caffeine levels we measured in decaff espresso coffee were substantially higher than expected, resulting in some “caffeine effect”.

### Electronic supplementary material

Below is the link to the electronic supplementary material.

Supplementary file1 (DOCX 12 kb)

## Data Availability

Raw data for all variables, including all time points, are available in an Excel table upon on request. Plasma caffeine assay details are available in the electronic supplementary material file.
