# Supplementary material for: Immediate effect of caffeine on sympathetic nerve activity: why coffee is safe? A single-centre crossover study
Source: Clin Auton Res. 2023 Aug 20;33(6):623–33. doi: 10.1007/s10286-023-00967-5 (PMC10751260; doi:10.1007/s10286-023-00967-5)
Supplement: Supplementary file 1 — Supplementary file1 (DOCX 12 kb) [file 10286_2023_967_MOESM1_ESM.docx]

Method for plasma caffeine assay

Appendix: plasma assay for caffeine levels

The internal standard β‑hydroxyethyl theophylline, 100 µL of 50 µmol/L, was added to 100 µL of plasma sample. The mixture was made alkaline with 100 µL of 1.0 mol/L sodium hydroxide and 250 mg of sodium chloride, then caffeine and the internal standard were extracted with 5.0 mL of chloroform containing 10% isopropyl alcohol. After shaking and centrifugation, an aliquot of the organic phase was filtered through a No 1 PS filter paper into a clean glass tube and evaporated to dryness under nitrogen. The residue was dissolved in 100 µL of mobile phase and 25 µL of the reconstituted sample was injected into the HPLC system.

HPLC analysis was performed on an Agilent 1200 series system equipped with a quaternary pump, a variable wavelength detector set at 275 nm, and an autosampler. Caffeine and the internal standard β‑hydroxyethyl theophylline were separated under isocratic elution using an Agilent XDB-C18 column, (1.8µm, 4.6 x 50 mm). The mobile phase was 10 mM sodium acetate and 12.6 mM sodium sulphate in water containing 13% acetonitrile, pH 5.05. The flow rate was 0.5 mL/min. Under the chromatographic conditions employed, the retention times were 2.7 and 3.8 min for the internal standard and caffeine respectively. The standard curve for caffeine was linear over the concentration range 0.30 - 150 µmol/L. The lower limit of quantification in plasma was 0.30 µmol/L. Intra- and inter-day coefficients of variation were < 10%.
